# Preoperative word-finding difficulties in children with posterior fossa tumours: a European cross-sectional study

**DOI:** 10.1007/s00381-023-06119-4

**Published:** 2023-09-08

**Authors:** K. Persson, D. Boeg Thomsen, Å. Fyrberg, C. Castor, M. Aasved Hjort, B. Andreozzi, P. Grillner, J. Kjær Grønbæk, J. Jakus, M. Juhler, C. Mallucci, R. Mathiasen, E. Molinari, B. Pizer, A. Sehested, A. Troks-Berzinskiene, K. van Baarsen, I. Tiberg, The CMS study group

**Affiliations:** 1 Department of Health Sciences, Lund University, Box 117, 221 00 Lund, Sweden; 2 Department of Nordic Studies and Linguistics, University of Copenhagen, Emil Holms Kanal 2, 2300 Copenhagen, Denmark; 3 Department of Neurosurgery, Copenhagen University Hospital Rigshospitalet, Blegdamsvej 9, 2100 Copenhagen E, Denmark; 4 Department of Speech and Language Pathology, University of Gothenburg, Medicinaregatan 11, 405 30 Gothenburg, Sweden; 5 Department of Pediatric Hematology and Oncology, St Olavs Hospital, Postboks 3250 Torgarden, 7006 Trondheim, Norway; 6 Department of Hematology/Oncology, Cell and Gene Therapy, Bambino Gesù Children’s Hospital, IRCCS, Rome, Italy; 7 Pediatric Oncology Unit, Astrid Lindgren’s Children’s Hospital, Karolinskavägen 6, 171 76 Stockholm, Sweden; 8 Department of Paediatrics and Adolescent Medicine, Copenhagen University Hospital Rigshospitalet, Blegdamsvej 9, 2100 Copenhagen E, Denmark; 9 WOW Speech Studio, Üllői út 189, Budapest, 1091 Hungary; 10 Department of Neurosurgery, Aarhus University Hospital, Palle Juul-Jensens, Boulevard 99, 8200 Aarhus, Denmark; 11 Department of Neurosurgery, Alder Hey Children’s NHS Foundation Trust, E. Prescot Road, Liverpool, L14 5AB UK; 12 University of Edinburgh, Old College, South Bridge, Edinburgh, EH8 9YL UK; 13 Department of Neurology, The Queen Elizabeth University Hospital, University of Glasgow, University Avenue, Glasgow, G12 8QQ UK; 14 University of Liverpool, Brownlow Hill, Liverpool, L69 3BX UK; 15 Department of Pediatrics, Lithuanian, University of Health Science, Mickeviciaus 9, 44307 Kaunas, Lithuania; 16 Princess Máxima Center for Pediatric Oncology, Heidelberglaan 25, 3584 CS Utrecht, The Netherlands

**Keywords:** Cerebellar mutism syndrome, Child language, Language impairment, Nordic-European CMS study, Posterior fossa tumour, Word-finding difficulties

## Abstract

**Purpose:**

Posterior fossa tumour surgery in children entails a high risk for severe speech and language impairments, but few studies have investigated the effect of the tumour on language prior to surgery. The current crosslinguistic study addresses this gap. We investigated the prevalence of preoperative word-finding difficulties, examined associations with medical and demographic characteristics, and analysed lexical errors.

**Methods:**

We included 148 children aged 5–17 years with a posterior fossa tumour. Word-finding ability was assessed by means of a picture-naming test, Wordrace, and difficulties in accuracy and speed were identified by cut-off values. A norm-based subanalysis evaluated performance in a Swedish subsample. We compared the demographic and medical characteristics of children with slow, inaccurate, or combined slow and inaccurate word finding to the characteristics of children without word-finding difficulties and conducted a lexical error analysis.

**Results:**

Thirty-seven percent (*n* = 55) presented with slow word finding, 24% (*n* = 35) with inaccurate word finding, and 16% (*n* = 23) with both slow and inaccurate word finding. Children with posterior fossa tumours were twice as slow as children in the norming sample. Right-hemisphere and brainstem location posed a higher risk for preoperative word-finding difficulties, relative to left-hemisphere location, and difficulties were more prevalent in boys than in girls. The most frequent errors were lack of response and semantically related sideordinated words.

**Conclusion:**

Word-finding difficulties are frequent in children with posterior fossa tumours, especially in boys and in children with right-hemisphere and brainstem tumours. Errors resemble those observed in typical development and children with word-finding difficulties.

**Supplementary Information:**

The online version contains supplementary material available at 10.1007/s00381-023-06119-4.

## Introduction

Central nervous system (CNS) tumours are among the most common paediatric cancer diagnoses and account for 3.2 new cases per 100,000 children [1]. Around half of the CNS tumours are located in the posterior fossa [2]. Surgery is a key therapy for posterior fossa tumours, but it entails the risk of a severe complication, cerebellar mutism syndrome (CMS), with persisting impairments in both speech and language [3]. Most studies in children with CMS focus on postoperative impairment but knowledge about preoperative ability is essential to evaluate to which degree deviations observed postoperatively were caused by surgery or were present before surgery. Preoperative language impairment, including word-finding difficulties, has been found to predict postoperative CMS [4, 5] but is still rarely investigated.

Word-finding ability is the ability to find the right word in the mental lexicon as we speak, quickly, and accurately [6]. In typical development, word-finding speed and accuracy increase with higher age [7–12] along with developments in children’s vocabularies which continuously increase in breadth, depth, and interconnectedness [10]. Word-finding difficulties are seen in children with different diagnoses — e.g. developmental language disorders and dyslexia — and they are the primary impairment in children classified as children with word-finding difficulties (WFDs) [6]. Word finding may be compromised by impairment of semantic processes (retrieving words as units of meaning), phonological processes (retrieving words as structures of speech sounds), motor planning (planning articulation), or motor execution (articulation) as well as by impairment in general processing speed [6, 13, 14]. These different sources of word-finding difficulties are partly reflected in distinct error profiles [6, 15], and they require different types of intervention [16]. For children with posterior fossa tumours, it is therefore important to clarify whether their error profiles resemble error profiles known from other diagnoses and/or from typical development.

The exact role of the cerebellum in successful word finding is unclear, but for each of the subprocesses of word finding, there is evidence suggesting that it may be involved [17–19]. One reason why cerebellar damage may compromise word finding is that the cerebellum is reciprocally linked to the neocortical areas involved in linguistic processing [20–24]. For most people, principal language-processing areas are located in the left cerebral hemisphere, and as each cerebral hemisphere sends and receives information to and from the contralateral cerebellar hemisphere [25], damage to the *right* cerebellar hemisphere can be expected to affect language. Indeed, for adults, neuroimaging and lesion studies converge on right-hemisphere cerebellar involvement in linguistic tasks [24, 26, 27], and for lexical retrieval specifically, there is evidence that right-sided cerebellar lesions disrupt naming and verbal fluency [22]. For children with posterior fossa tumours, there is preliminary evidence for right-hemisphere location increasing the risk of linguistic impairment after surgery [28, 29].

*Before* surgery, one study found linguistic impairment in more than a quarter of children with posterior fossa tumours, and word-finding difficulties were significantly associated with brainstem involvement, invasion of the right dentate nuclei, and severe hydrocephalus [30]. Effects of right-hemisphere vs. left-hemisphere location were not investigated.

In the current study we ask:

1. How many children with posterior fossa tumours experience word-finding difficulties, defined as slow and/or inaccurate word finding, before surgery?
2. How are preoperative word-finding difficulties associated with demographic and medical characteristics in children with posterior fossa tumours? For location, we hypothesise that right-hemisphere and brainstem locations increase the risk of word-finding difficulties relative to left-hemisphere location. For the remaining parameters, the analysis is explorative.
3. Do the word-finding errors we see in children with posterior fossa tumours resemble or diverge from the types known from other child populations with word-finding difficulties and/or from typical development?

## Methods

### Study design and setting

This crosslinguistic, cross-sectional study is part of an observational cohort study, the Nordic-European CMS study, described in detail elsewhere [31, 32]. We included children aged 5–17 years with a tumour in the posterior fossa who were treated at centres between 2014 and 2022 in Sweden, Denmark, Norway, the UK, Hungary, Italy, the Netherlands, and Lithuania. The study was approved by regional and national ethics committees. The participants were approached with information about the study by the child’s physician, and legal caregivers provided written informed consent for all children.

### Participants

**Fig. 1:**
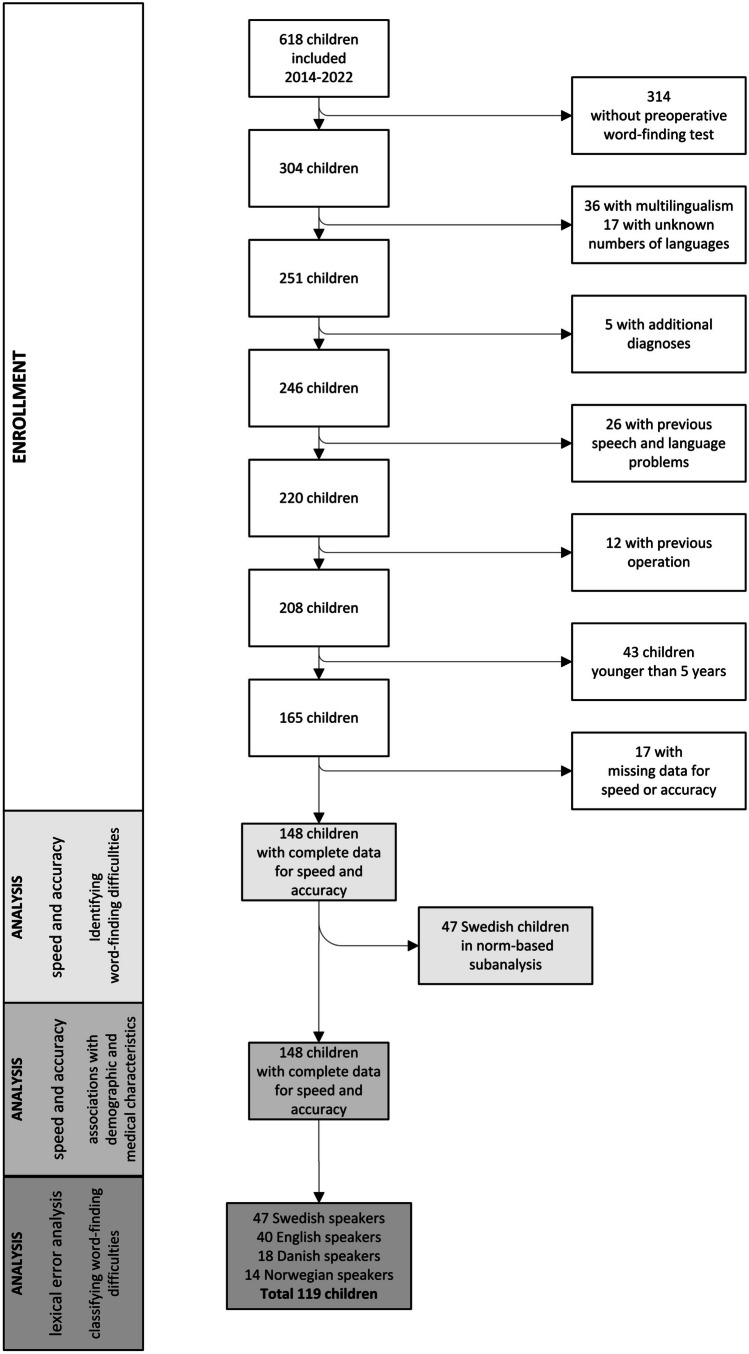
The inclusion process and the children included in each analysis

Between 2014 and 2022, 618 children younger than 18 years were included in the Nordic-European CMS study. We excluded multilingual children, children with additional diagnoses, previously reported speech and language disturbances and previous tumour surgery. Due to lack of norm data from children younger than five years for the assessment tool, we excluded children younger than 5 years old. We discarded data from children whose data for speed and/or accuracy were invalid due to experimenter error and included 148 children with complete data for both speed and accuracy. In a subanalysis utilising Swedish word-finding norms, 47 Swedish children were included. In a lexical error analysis, children from Sweden, Great Britain, Denmark, and Norway were included, in total 119 children. Figure 1 presents the inclusion process and the numbers of children included in each analysis.

### Materials

To assess word-finding ability, we used the 25-item picture-naming test Wordrace which was designed for the Nordic-European CMS study as an instrument for assessing word-finding speed and accuracy [31, 33]. Details on Wordrace can be found in the online supplemental materials. Normative data is only available for Swedish, where a Master’s thesis based on 299 typically developing children aged 5–15 years showed a negative correlation between age and speed in seconds (as children get older, they take fewer seconds to complete the task). Most of the children had no errors (named all items correctly). The Swedish norms are used in the Swedish subanalysis to evaluate word-finding performance in children with posterior fossa tumours.

### Data collection

Wordrace was administered at the hospitals by a physician, a nurse or a speech and language pathologist. Pictures were presented one at a time, on screen or on paper, and children were instructed to name the pictures as rapidly as possible. The test leader switched/browsed to the next picture as soon as a picture was named. If children failed to name a picture, they were not given any cues, and the next picture was shown after 5 s. The test procedure was audio recorded.

Preoperative demographic data on age, sex, dysarthria, hydrocephalus, oculomotor abnormalities, tumour location, and tumour histology were collected via study protocols by clinicians. Dysarthria was reported in four grading categories from normal to absent/unintelligible speech and oculomotor abnormalities were reported in four grading categories from normal to nystagmus. Hydrocephalus was reported as absent or present. Tumour location was reported postoperatively and could include more than one site in the posterior fossa: left hemisphere, right hemisphere, cerebellar vermis, fourth ventricle, and/or brainstem involvement. Tumour histology was reported as pilocytic or pilomyxoid astrocytoma (hereafter: pilocytic astrocytoma), medulloblastoma, ependymoma, atypical teratoid rhabdoid tumour, or other.

### Analysis

Wordrace was scored by speech analysts in each country.^1^ Two parameters were scored: speed (in seconds) and accuracy (number of correctly named pictures). Alternative responses were reported. We identified preoperative word-finding difficulties by means of cut-off values based on Swedish norms [8], results from other studies [7, 9, 10, 34] and clinical experience. Because the assessments were conducted in different formats (screen/paper), in different languages and by different professions, and because the sample included children with dysarthria, we set a generous cut-off for speed. This ensured specificity (i.e. only identifying children with genuinely slow word finding), but it compromised sensitivity (i.e. we cannot be sure that children *not* identified by our cut-off have normal word-finding speed). For speed, the cut-offs were ≥ 70 s for 5–9 years, ≥ 60 s for 10–12 years, and ≥ 55 s for 13–15 years. For accuracy, the cut-offs were ≥ 23 for 5–9 years and ≥ 24 for 10–17 years. A subanalysis was conducted comparing speed and accuracy with the Swedish norms.

For the Scandinavian languages and English a lexical error analysis was conducted, classifying alternative responses in 13 categories, in accordance with earlier research [11]. To examine interrater reliability, two speech and language therapists with more than 15 years of clinical experience categorised the alternative responses independently with agreement on 86% of the 92 alternative responses. Cases with disagreement were discussed, and consensus reached.

Dysarthria and oculomotor abnormalities were classified as absent or present. For tumour type, we used the classification from the study protocol (see “Data collection” section), with the exception that atypical teratoid rhabdoid tumour (*n* = 1) was categorised as “other”.

Tumours were reported as being in numerous combinations of locations, and we grouped these in five categories:

1. Left cerebellar hemisphere (including max. one other location: vermis, fourth ventricle or brainstem)
2. Right cerebellar hemisphere (including max. one other location: vermis, fourth ventricle or brainstem)
3. Vermis (including max. one other location: fourth ventricle or brainstem)
4. Brainstem (including max. one other location: fourth ventricle)
5. Other (tumour extending into three or more locations, fourth ventricle)

These broad categories were used for statistical analysis, but for transparency, we specify subcategories of location in the tables.

## Results

Demographics are shown in Table 1. Most children were speakers of a Scandinavian language (52%) or English (29%), and 6% had preoperative dysarthria.

**Table 1:** Demographic data

	**Participants aged 5–17 (*N* = 148)**
Age categories, years, *n* (%)	
(median, Q1,Q3)	9:8, 7:2, 12:9
5–9	77 (52)
10–12	35 (24)
13–17	36 (24)
Gender, *n* (%)	
Female	70 (47)
Male	78 (53)
Language, *n* (%)	
Swedish	47 (32)
English	40 (27)
Danish	18 (12)
Norwegian	14 (9)
Hungarian	12 (8)
Italian	6 (4)
Dutch	5 (3)
Lithuanian	6 (4)
Dysarthria, *n* (%)	
Present	8 (5)
Absent	120 (81)
Unknown	20 (14)
Tumour location, *n* (%)	
Left cerebellar hemisphere	25 (17)
LCH	17 (11)
LCH + VR/FV	6 (4)
LCH + BS	2 (1)
Right cerebellar hemisphere	34 (23)
RCH	22 (15)
RCH + VR/FV	8 (5)
RCH + BS	4 (3)
Vermis	25 (17)
VR	16 (11)
VR + FV	6 (4)
VR + BS	3 (2)
Brainstem	17 (11)
BS	13 (9)
BS + FV	4 (3)
Other^a^	47 (32)
Tumour histology, *n* (%)	
Pilocytic astrocytoma	62 (42)
Medulloblastoma	47 (32)
Ependymoma	12 (8)
Other	14 (9)
Unknown	13 (9)
Hydrocephalus, *n* (%)	
Present	89 (60)
Absent	53 (36)
Unknown	6 (4)
Oculomotor abnormalities, *n* (%)	
Present	39 (26)
Absent	88 (59)
Unknown	21 (14)

The data are in *n* (%)

*LCH* left cerebellar hemisphere, *RCH* right cerebellar hemisphere, *BS* brainstem, *VR* vermis, *FV* fourth ventricle

^a^Other: tumour extending into three or more locations and tumour located in only forth ventricle

### Identification of preoperative word-finding impairment

Thirty-seven percent (*n* = 55) of the children were identified as having slow word-finding, defined as exceeding our cut-offs for speed (see Table 2). Four of them had dysarthria.

**Table 2:** The distribution of children’s performance in speed and accuracy based on cut-off values with language, test format and motor speech status specified

Speed			Accuracy	
	Fast word-finding	Slow word-finding	Accurate word-finding	Inaccurate word-finding
All	93 (63)	55 (37)	113 (76)	35 (24)
Language				
Swedish	27 (57)	20 (42)	36 (83)	11 (23)
English	27 (68)	13 (33)	27 (68)	13 (33)
Danish	16 (89)	2 (11)	14 (78)	4 (22)
Norwegian	9 (64)	5 (36)	13 (93)	1 (7)
Hungarian	5 (42)	7 (58)	5 (42)	7 (58)
Italian	5 (83)	1 (17)	6 (100)	0 (0)
Lithuanian	1 (17)	5 (83)	5 (83)	1 (17)
Dutch	3 (60)	2 (40)	5 (100)	0 (0)
Test format				
Paper	29 (49)	30 (61)	42 (71)	17 (29)
Screen	64 (72)	25 (28)	71 (80)	18 (20)
Dysarthria				
Yes	4 (50)	4 (50)	5 (63)	3 (38)
No	73 (61)	47 (39)	95 (79)	25 (21)
Unknown	16 (80)	4 (20)	13 (65)	7 (35)

The distribution is in *n* (%)

Twenty-four percent (*n* = 35) of the children were identified as having inaccurate word-finding, defined as naming fewer of the pictures correctly than required by our cut-offs for accuracy (see Table 2). Three of them had dysarthria.

**Fig. 2:**
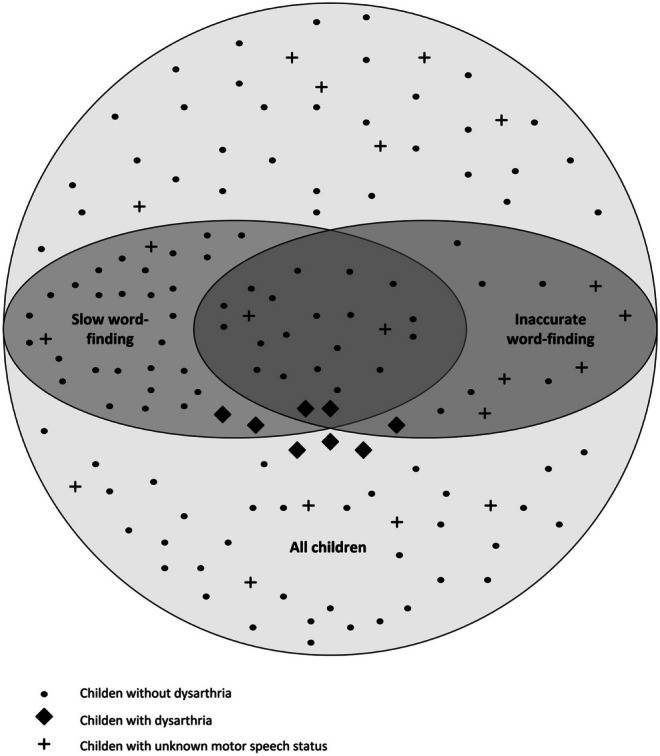
Distribution of children with and without impairment in speed and/or accuracy. Motor-speech status is specified

Accuracy and speed could be independently impaired but 16% (*n* = 23) of the children had problems in both speed and accuracy, as illustrated by Fig. 2.

**Fig. 3:**
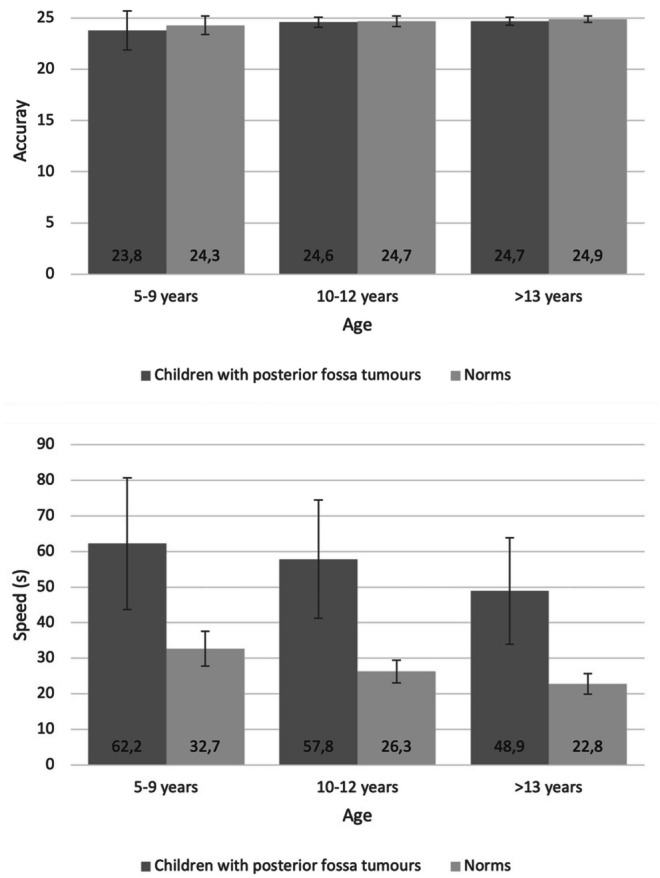
Performance in speed and accuracy in Swedish children with posterior fossa tumours compared with the Swedish norms (means and standard deviations). Speed is in seconds and accuracy in number of correct responses

For the subset of Swedish children with posterior fossa tumours, we compared performance with norms from typically developing children (5–9 years: *n* = 206, 10–12 years: *n* = 57, 13–15 years: *n* = 36). As demonstrated in Fig. 3, there were radical differences in mean speed, with children with posterior fossa tumours taking approximately twice the time compared to typically developing children and even the oldest children with tumours being much slower than the youngest children in the norming sample. For accuracy, in contrast, the differences were negligible.

#### Associations with demographic and medical characteristics

We compared the demographic and medical characteristics of children without word-finding difficulties, with slow word finding, with inaccurate word finding, and with both slow and inaccurate word finding (Table 3). For the hemispheric tumours central to our hypothesis, there was a clear lateralisation difference between the groups without word-finding problems and with combined slow and inaccurate word finding: for children without word-finding problems, 20% had the tumour located in the left hemisphere, 16% in the right, whereas for children with combined word-finding problems, 4% had the tumour located in the left hemisphere, and more than seven times as many, 30%, in the right. The distribution of hemispheric tumours differed significantly between these two groups (Fisher’s exact: *p* < 0.05). Brainstem location also increased the risk for severe impairment relative to left-hemisphere location, with 17% brainstem tumours in the group with combined word-finding difficulties compared to 7% in the group without word-finding difficulties (Fisher’s exact: *p* < 0.05). For children with a right-hemisphere or brainstem tumour, the odds of having combined word-finding difficulties were 2.99 times those of children with all other tumour locations (95% CI: 1.1383–7.8607, *p* < 0.05). Vermian tumours were frequent in children with combined difficulties (26%), but not significantly more than in children without difficulties (19%).

**Table 3:** Demographic and medical characteristics of children with no difficulties, slow word finding, inaccurate word finding, and children with both slow and inaccurate word finding

Parameters	No word-finding difficulties	Slow word-finding	Inaccurate word-finding	Both slow and inaccurate word-finding
	81 children	55 children	35 children	23 children
Sex				
Male	37 (46)	29 (53)	21 (60)	16 (70)
Female	44 (54)	26 (47)	14 (40)	7 (30)
Age				
Mean	9:8	10:5	10:4	9:8
Median (range)	9:2 (5–17:9)	10:4 (5:3–17:9)	10:9 (5:3–16:1)	10:3 (5:8–16:1)
Tumour histology, *n* (%)				
Pilocytic astrocytoma	34 (48)	23 (43)	19 (58)	14 (61)
Medulloblastoma	26 (37)	17 (32)	9 (27)	6 (26)
Ependymoma	8 (11)	3 (6)	2 (6)	1 (4)
Other	3 (4)	10 (19)	3 (9)	2 (9)
Unknown	10	2	2	0
Tumour location, *n* (%)				
Left cerebellar hemisphere	16 (20)	8 (15)	2(6)	1 (4)
LCH	11 (14)	5 (9)	2 (6)	1 (4)
LCH + VR/FV	3 (4)	3 (5)	0 (0)	0 (0)
LCH + BS	2 (2)	0 (0)	0 (0)	0 (0)
Right cerebellar hemisphere	13 (16)	18 (33)	10 (29)	7 (30)
RHC	12 (15)	8 (15)	4 (11)	2 (9)
RCH + VR/FV	1 (1)	7 (13)	3 (9)	3 (13)
RCH + BS	0 (0)	3 (5)	3 (9)	2 (9)
Vermis	15 (19)	8 (15)	8 (23)	6 (26)
VR	9 (11)	6 (11)	5 (14)	4 (17)
VR + FV	4 (5)	2 (4)	2 (6)	2 (9)
VR + BS	2 (2)	0 (0)	1 (3)	0 (0)
Brainstem	6 (7)	10 (18)	5 (14)	4 (17)
BS	4 (5)	8 (15)	5 (14)	4 (17)
BS + FV	2 (2)	2 (4)	0 (0)	0 (0)
Other^a^	31 (38)	11 (20)	10 (29)	5 (22)
Dysarthria, *n* (%)				
Present	3 (4)	4 (8)	3 (11)	2 (10)
Absent	67 (96)	47 (92)	25 (89)	19 (90)
Unknown	11	4	7	2
Hydrocephalus, *n* (%)				
Present	48 (62)	34 (65)	20 (57)	13 (57)
Absent	30 (38)	18 (35)	15 (43)	10 (43)
Unknown	3	3	0	0
Oculomotor abnormalities, *n* (%)				
Present	18 (26)	21 (42)	9 (32)	9 (45)
Absent	51 (74)	29 (58)	19 (68)	11 (55)
Unknown	12	5	7	3

The data are in *n* (%). The unknown data are not included in the percent calculations

*LCH* left cerebellar hemisphere, *RCH* right cerebellar hemisphere, *BS* brainstem, *VR* vermis, *FV* fourth ventricle

^a^Other tumour extending into three or more locations and tumour reported as located in only fourth ventricle

There was a significant difference between sex distribution in children with both slow and inaccurate word finding (70% males) and children without word-finding difficulties (46% males; χ2 = 4.0897, *p* < 0.05), and the odds of boys having combined difficulties were 2.72 times those of girls (95% CI: 1.0100–7.3149, *p* < 0.05). Oculomotor abnormalities were (non-significantly) more frequent in the group with slow and inaccurate word finding (45%) than in the group without word-finding difficulties (26%, *χ2* = 2.6244, *p* = 0.10523). There were no significant effects of tumour histology, dysarthria or hydrocephalus.

### Classification of word-finding errors

In 92 cases (3% of all responses), the child’s response was not in accordance with the target word, and in 41 of these, the child did not produce any word (“no response/aborted response”). In the remaining 51 cases, the child produced an alternative response. Among a variety of alternative response types (see Table 4), choosing a sideordinated word, i.e. a semantically closely related word (e.g. “pear” for “apple”), was the most frequent type of lexical error (*n* = 28).

**Table 4:** The frequency distribution of responses linguistically categorised in Wordrace

Total responses	2975		
In accordance with target word	2883		
*Not* in accordance with the target word	92		
Types of errors	Number of errors	Percentage of all errors	Percentage of all responses
No response/aborted response	41	45	1.4
Related sideordinated word^a^	28	30	1.0
Contextual association	6	7	0.2
Unrelated	7	8	0.2
Visual association	5	5	0.2
Subordinated word	1	1	0.0
Unrelated sideordinated word^b^	2	2	0.1
Superordinated word	1	1	0.0
Neologism	1	1	0.0

^a^Semantically closely related

^b^Semantically in the same category but not closely related

## Discussion

Word-finding difficulties are commonly reported after posterior fossa surgery, and this crosslinguistic study contributes new knowledge about the effect of posterior fossa tumours on word-finding abilities prior to surgery. Analysing word-finding performance in eight different languages, we found that 37% of the children were slower than expected for their age, 24% were less accurate, and 16% were both slower and less accurate. A subanalysis demonstrated that the subset of Swedish children was twice as slow as typically developing children. As predicted by functional cerebellar topography, right-hemisphere location posed a higher risk for preoperative word-finding difficulties than left-hemisphere location. Brainstem location was also associated with preoperative word-finding difficulties, as was male sex. The most frequent error types were lack of response and semantically related sideordinated words.

### Preoperative word-finding difficulties: prevalence and factors

Our results replicate a previous finding by di Rocco and colleagues, who found that preoperative word-finding difficulties are frequent in children with posterior fossa tumours [30]. In their Italian sample, word-finding difficulties were present in 26.8%, i.e. a similar proportion to the 24% presenting inaccurate word finding in our crosslinguistic sample. We found a higher proportion of children with slow word finding (37%), but di Rocco and colleagues did not specify impairment in speed.

Based on evidence from adults [22, 26, 27] and children [28, 29], we hypothesised that tumour location in the right cerebellar hemisphere would increase the risk of word-finding difficulties relative to left-hemisphere location. Our results provided support for this hypothesis and thus support theories of functional cerebellar topography [26, 27] and cerebellar language lateralisation [24]. We also replicated a previous finding that brainstem involvement increases the risk of preoperative word-finding difficulties [30]. Our analyses of the remaining parameters were explorative, and we found no significant effects of tumour histology, dysarthria, or hydrocephalus. At first sight, it may be surprising that we found no differences between tumour types given that medulloblastoma is a known risk factor for postoperative cerebellar mutism [32]. In our preoperative sample, the share of children with medulloblastoma or ependymoma was slightly *lower* in children with both slow and inaccurate word finding, whereas the share of children with pilocytic astrocytoma was slightly higher, but this difference was non-significant. Our results may indicate that the negative effect of medulloblastomas depends on events associated with their surgical removal, not on effects of the tumour itself. We did observe a significant effect of sex, with a higher prevalence of boys with both slow and inaccurate word finding, aligning with studies of other populations where the proportion of language-impaired males is typically also higher [35, 36].

### Word-finding errors

Two types of errors accounted for most of the errors in our sample: (1) failure to produce any word and (2) semantically related sideordinated words (such as “swan” for “goose”). This pattern resembles the one seen in both typically developing children and children with WFDs [7, 11, 15]. The many semantically related errors reveal that the mental lexicons of children with posterior fossa tumours are still robustly organised in categories. There may be many explanations why children do not settle on the right word even when searching in the correct semantic category, for instance, underspecified representations of word meanings and/or slower processing speed [6]. The semantic errors indicate impairment at the level of semantic retrieval, and for individual children with many such errors, semantic intervention developed for children with WFDs may prove useful [16]. Errors involving failure to produce a word are more difficult to interpret, as they may arise from several reasons, including semantic, phonological, or speed-related difficulties, and may also reflect behavioural styles [11, 15].

### Limitations and future directions

Wordrace lacks norms except for Swedish, so to identify word-finding difficulties, we created cut-off values, based on Swedish norms, other studies, and clinical experience. For speed, the cut-off values were generous, to ensure test specificity, but test sensitivity is likely to have been compromised, and we may not have identified all children with slow word-finding speed. We therefore consider this study a first step and hope that future studies will be able to identify word-finding impairment more precisely using age- and language-specific norms. We strongly encourage norming of Wordrace in more languages because it is a highly useful word-finding test, posing minimal demands on executive control and lexicon, contrary to other instruments, such as the Boston Naming Test (BNT) [37] and verbal fluency tests [38–40].

As for tumour location, the study protocol provided information in the sense of listing all areas encroached on by the tumour, but it did not provide information about *main* location or about how much the tumour infiltrated any of the locations listed. Such information would make the analysis of associations between tumour location and word-finding difficulties more precise. Within the larger Nordic-European CMS study, MRI data have been collected, and they will provide the basis for future studies with more granular location information.

Our analysis of medical characteristics also revealed a potential source of error to be checked in future studies: oculomotor abnormalities were overrepresented in children with combined slow and inaccurate word finding. While the difference in distribution was non-significant, we cannot exclude that some children identified as having word-finding problems were in fact impeded by impaired visual processing of the visual test stimuli.

Assessing preoperative word-finding difficulties provides a baseline for evaluating postoperative word-finding difficulties, making it possible to examine to which degree these reflect exacerbation or improvement of word-finding abilities prior to surgery. A predictive relationship has been found between preoperative language impairment and postoperative CMS [4], and in future studies, it will also be important to investigate whether children with preoperative word-finding difficulties are at a higher risk of developing postoperative word-finding difficulties and CMS. For children whose preoperative word-finding difficulties do not resolve after tumour resection, future studies should investigate whether they will benefit from word-finding interventions developed for children with WFDs. Supporting children’s word-finding ability is important, as word-finding difficulties affect well-being and participation in everyday life.

### Supplementary Information

Below is the link to the electronic supplementary material.

Supplementary file1 (DOCX 5071 KB)

## Data Availability

Due to ethical considerations, data associated with this paper cannot be accessed.

## Footnotes

1 With the exception of English, which was scored centrally.
