# Supplementary material for: Preoperative word-finding difficulties in children with posterior fossa tumours: a European cross-sectional study
Source: Childs Nerv Syst. 2023 Sep 8;40(1):87–97. doi: 10.1007/s00381-023-06119-4 (PMC10761395; doi:10.1007/s00381-023-06119-4)
Supplement: Supplementary file 1 — Supplementary file1 (DOCX 5071 KB) [file 381_2023_6119_MOESM1_ESM.docx]

# **Supplemental Materials: Wordrace**

Wordrace [1] is a word-finding test designed for measuring speed and accuracy of word finding in children and adolescents. It was developed for the Nordic-European Study of the Cerebellar Mutism Syndrome (CMS) in Children with Brain Tumours of the Posterior Fossa by a Swedish speech-language therapist and a pediatrician specialized in neuro-oncology.

At the time of writing (June 2023), Wordrace has only been normed in Swedish (based on the performance of 299 typically developing Swedish children aged 5-15 years), and in the Swedish norm sample, high test-retest reliability was found for both speed (*r=*.894**, *p*=.000) and accuracy (*r*=.627****, *p*=.000) [2]. Normative data from other languages are needed to establish with certainty what counts as typical vs. atypical performance in children speaking other languages. Normative studies are currently planned for Danish, Dutch, English and Italian.

This document presents basic information about the test, listing the 25 target words and accepted alternatives, giving examples of the stimulus pictures, and presenting the test developers’ instructions for administration as well as the coding guidelines we developed, including information on cases of experimenter error where data have to be discarded as invalid.

**Target words and accepted alternatives**

| **Item** | **Target**(Target words are in bold, followed by accepted alternative responses) |
| --- | --- |
| 1 | **Duck, goose** |
| 2 | **Cow** |
| 3 | **Orange**, clementine, mandarin, tangerine |
| 4 | **Key** |
| 5 | **Scissors** |
| 6 | **Dice** |
| 7 | **Bread**, **loaf** |
| 8 | **Apple** |
| 9 | **Banana** |
| 10 | **Burger** |
| 11 | **Ice cream**, cone |
| 12 | **Hammer** |
| 13 | **Umbrella** |
| 14 | **Glasses**, sunglasses |
| 15 | **Balloons** |
| 16 | **Cake** |
| 17 | **Gift**, **present** |
| 18 | **Rooster**, **cockerel**, hen, chicken |
| 19 | **Frog**, toad, lizard, gekko |
| 20 | **Bag**, **handbag** |
| 21 | **Car** |
| 22 | **Boat, ship**, ferry |
| 23 | **Cat** |
| 24 | **Star** |
| 25 | **Flower** |

**Examples of stimulus pictures**

Item 2: *cow*

Item 4: *key*

**

Item 8: *apple*

Item 15: *balloons*

**

Item 18: *rooster*, *cockerel*

# **Instructions for administration of Wordrace**

1. Make sure that the child is settled and attending.

2. Sit next to the child in front of the computer/iPad.

3. Give the child the following instruction:

*Now I'll show you some pictures of ordinary things, one at a time. Your job is to name the picture using only one word, for instance, “dog” or “book”. The game is to find the words as fast as possible. If you can´t find the word we´ll just move on to the next picture.*

4. Ensure the recording equipment is switched on. Say the child's name and the date.

Then say:

*Now it´s time to start. I will show you the first picture on three.*

*Are you ready? One, two, THREE* (show picture 1 the moment you say *three*).

5. Show the pictures. If the child can’t name the picture within 5 seconds, go on to the next picture. It is important to refrain from providing any verbal or nonverbal cues prior to the

child’s response (including *What is this?*).

6. When the child has completed the test, say *Good job!* and then turn off the recorder.

**Instructions for coding of Word Race**

Two of the categories in the CMS coding sheet depend on scoring of Word Race:

1. Word Race: Total test-time (Word-finding abilities)
2. Word Race: Number of correctly named words (Word-finding abilities)

**Coding steps**

1. Calculate total test-time:
2. Start the timekeeping when the first picture is shown.
3. Stop when the last picture is named.
4. Register the time in seconds, max. 125 seconds. (Total test-time)
5. Calculate number of correctly named words:

- Correctly named items earn 1 point.
- Incorrectly named items earn 0 points (note the answer).
- Items not named (within 5 seconds) earn 0 points.
- Register the number of correctly named words, max. 25 (Number of correctly named words)

**Invalid responses**

1. If the child has been given more than 125 seconds, the test results are not valid, and neither test time nor number of correctly named words is registered. Write N/A in the boxes for registering total test-time and number of correctly named words, and make a note in the comments box stating why.
2. If the child has not seen all 25 test pictures within 125 seconds, the test results are not valid. Neither test time nor number of correctly named words is registered. Write N/A in the boxes for registering total test-time and number of correctly named words, and make a note in the comments box stating why.
3. If the test leader has given the child more than 5 seconds to respond to a picture, and the child names the picture correctly *after* 5 seconds, the child’s response to that picture counts as not named and earns 0 points. Make a note in the comments box stating why.
4. If the test leader or caregiver helps the child in any way to give a correct answer *within* the first 5 seconds and before the child has responded, the child’s guided response to that picture does not count and is scored as N/A. If help is given *after* 5 seconds, the child’s response to that picture counts as not named and earns 0 points. In either case, make a note in the comments box stating why.
5. If the test has been interrupted (e.g. by a person entering the room), total test-time is not registered (write N/A in the relevant box), but the number of correctly named words can still be registered. Make a note in the comments box stating why test-time is not registered.

**References**

1. Grillner P, Zetterqvist B. Wordrace. 2014.

2. Persson K. Word finding speed develops with age: Normative data for Wordrace in children and adolescents: Master thesis, University of Gothenburg; 2020.
